# Knowledge, attitude, and practice of breast Cancer among nurses in hospitals in Asmara, Eritrea

**DOI:** 10.1186/s12912-018-0300-4

**Published:** 2018-07-31

**Authors:** Amanuel Kidane Andegiorgish, Eyob Azeria Kidane, Merhawi Teklezgi Gebrezgi

**Affiliations:** 1 Department of Epidemiology and Biostatistics, Asmara College of Health Sciences, School of Public Health, P.O.Box: 8566, Asmara, Eritrea; 2 Department of Epidemiology, Robert Stempel College of Public Health and Social Work, Florida International University, 11200 SW 8th St, Miami, FL 33199 USA

**Keywords:** Breast cancer, Knowledge, Attitude, Practice, Nurse, Asmara, Eritrea

## Abstract

**Background:**

Breast cancer accounted for 1.03% of all deaths in 2014 in Eritrea. Yet the knowledge, attitude, and practice (KAP) of the population in general or the health personnel in the country in relation to the disease, remains unknown. Hence, this study was designed to assess the KAP regarding breast cancer among female nurses working in ten hospital wards in Asmara, Eritrea.

**Methods:**

This was a cross-sectional study conducted among 414 nurses. Descriptive statistics, t-test, and ANOVA were used to evaluate the KAP of the nurses.

**Results:**

Nurses’ knowledge about the possible risk factors of breast cancer was low but the nurses knew the signs and symptoms of breast cancer since each sign or symptom was mentioned by > 50% of them. The practice of breast cancer screening, however, was low (only 30 and 11.3% practiced clinical breast examination and mammography respectively). Respondents’ family history of breast cancer, having breast problems, their professional level and unit where they worked were associated with the KAP of nurses about breast cancer.

**Conclusion:**

Training programs could help to increase the nurses’ knowledge about the risk factors of breast cancer and practice of breast cancer screening. This could also help to increase the knowledge of the public about breast cancer.

## Background

Breast cancer is a major, life-threatening, public health concern. Long-term increase in the incidence of the disease has been observed in both developed and developing countries [1]. It is the most common cause of cancer mortality among women, accounting for 16% of cancer deaths in adult women [2].

In Eritrea, 370 women died from breast cancer in 2014 accounting for 1.03% of total deaths in that year giving an age-adjusted death rate of 21.40 per 100,000 of population [3]. Other source shows similar figures, with increasing rates in recent years in the country [4]. All malignant neoplasms of the breast in all age groups of women reported from all health facilities in Eritrea in the last 13 years (2004–2016) show a steady growth, with minor fluctuations in 2005 and 2009 (Health Information Management System of the Ministry of Health).

Breast cancer risk factors include increased age, genetic mutation, early menstrual period, late or no pregnancy, starting menopause after age 55, not being physically active, being overweight or obese after menopause, having dense breast, using combination hormone therapy, taking oral contraceptives, personal history of breast cancer, personal history of certain non-cancerous breast diseases, a family history of breast cancer, previous treatment using radiation therapy and drinking alcohol [5]. Adequate knowledge about the signs and symptoms and early breast cancer detection through breast self-examination (BSE) or clinical breast examination (CBE) or mammogram, is crucial to reducing breast cancer-related morbidity and mortality.

Information is needed to identify the underlying factors that might influence nurses’ own practice of early detection methods of breast cancer. Empowering nurses with information about early detection methods and their related benefits could help in advancing their skills in performing breast self-examination and expanding their role as client educators [6]. Education and awareness need to be culturally appropriate and targeted towards the relevant population, because this may contribute towards an increase early presentation so that highest benefit can be gained [7, 8]. Health care providers, especially those who come in regular contact with women, can play an important role in providing the information regarding breast cancer [9].

Several cross-sectional studies on Knowledge Attitude and Practice (KAP) of breast cancer have been done among nurses in Nigeria [10–13] Ethiopia [14], Turkey [15] Jordan [16, 17], India [18], United Arab Emirates [19], Saudi Arabia [20] and Singapore [21]. Nurses KAP on breast cancer was ranging from relatively low to as high as 100%. There were varying responses in attitude, knowledge of breast cancer screening methods and practice.

Previously, studies on the clinical aspects of breast cancer have been conducted in Eritrea [22–24]. Yet, no published study is available about the KAP of breast cancer among nurses in the country. The aim of this study was, therefore, to assess the (1) nurses’ knowledge of breast cancer risk factors, (2) nurses’ knowledge of breast cancer signs and symptoms, and (3) nurses’ knowledge and practice of breast cancer examinations among female nurses working in ten hospital wards in Asmara. The information obtained could help to initiate interventions to address the gaps in KAP of nurses towards breast cancer. Ultimately, it is hoped that an improved KAP of nurses will contribute towards educating the public about the risk factors and early detection of breast cancer and, thereby, reduce morbidity and mortality due to breast cancer in the population.

## Methods

### Study design and study population

This was a cross-sectional study designed to assess the KAP of nurses on breast cancer. It was conducted among nurses whose qualifications included certificate, diploma, and degree and who were working in ten hospital wards in Asmara, the capital of Eritrea. All the nurses in the selected health facilities and wards were invited to participate in the study. Majority of the nurses working in the surveyed hospital wards were females.

### Data collection

Knowledge of breast cancer among the participants was assessed based on knowledge on risk factors of breast cancer, signs and symptoms of breast cancer and knowledge on BSE, CBE, and mammography. The assessment was done by scoring breast cancer knowledge computed by giving “1” to the correct answer, and “0” for the wrong and ‘do not know’ answers. Furthermore, a percent knowledge index (PKI) was calculated for each nurse by summing the number of correct answers for all the variables and calculating the percentage of the correct answers. Furthermore, attitude was assessed by asking respondent about their opinions in regards to whether breast cancer is curable, how they would feel if they develop breast cancer and what they would do if they develop BC. Practice was assessed on what respondents do to detect early symptoms and signs of BC.

### Data analysis

Data was collected by a pretested, questionnaire. List of study subjects was obtained initially from the Human Resource Management (HRM) office of the studied hospital wards, and after verbal consent was obtained, an interviewer guided self-administered questionnaire was used to collect the data. Collected data were checked for errors and data entry was completed using census survey processing (CSPro) software. Data were analyzed using the statistical package for social sciences (SPSS version 20) software. Descriptive statistics were used for data presentations. Association between categorical variables were explored using Chi-square. Quantitative analysis was performed using t-test for binary variables and ANOVA for more than two variables. *P* < 0.05 was considered statistically significant.

### Ethical approval

The study was approved by the Research and Ethics Committee of Asmara College of Health Sciences and Eritrean National Commission for Higher Education. An official letter was sent to concerned hospital directors and oral informed consent was obtained from the study subjects prior to questionnaire administration.

## Results

A total of 427 nurses were approached to participate in this study. The complete response rate was 97% and so a total of 414 nurses’ responses were included in the data analysis. The mean age of participants was 33.85 ± 11.60 (min 19 and max 69 years). Nearly half (48.6%) of the participants were associate nurses. Similarly, half of the respondents (51%) were unmarried. More than nine-tenths of study participants had no family history of breast cancer or no personal history of breast problems (Table 1).

**Table 1:** Socio-demographic characteristics of nurses working in ten health facilities in Asmara, Eritrea

Variable	Frequency	Percent
Age		
19–29	222	53.6
30–39	65	15.7
40–49	63	29.7
> =50	64	31.7
Marital Status		
Single	212	51.2
Ever married	202	48.8
Religion		
Muslim	35	8.5
Christian	379	91.5
Family Hx of Breast Cancer		
Yes	32	7.7
No	382	92.3
Having breast problem		
Yes	20	4.8
No	394	95.2
Professional Level		
Associate Nurse	201	48.6
Registered Nurse	170	41.1
BSN	43	10.4
Unite of Work		
Orotta MCH	38	9.1
Private hospital	44	10.6
Medical wards	162	38.9
Surgical Ward	50	12.0
Community hospitals	82	19.7
Ophthalmic hospital	40	9.6

*BSN* Bachelors of Science in Nursing, *MCH* Maternity Child Health

### Knowledge about risk factors of breast cancer

Table 2 shows the responses of the nurses about the risk factors of breast cancer. About 78% of the participants mentioned that smoking is a risk factor for breast cancer. Similarly, 76% stated that breastfeeding decreases the risk of breast cancer and 62% stated that drinking alcohol is a risk factor. About two thirds (69%) agreed that breast cancer could be hereditary and 66% thought that its risk increases with advancing age. Approximately one-third of the nurses stated that having a first child when over 30 is a possible risk factor for breast cancer.

**Table 2:** Knowledge about risk factors of breast cancer among nurses in Asmara, Eritrea

Questions	Yes	Percentage (%)	95% Confidence Interval
Does breast cancer risk increases with advancing age	276	66.35	[61.79–70.90]
Is breast cancer hereditary	287	68.99	[64.53–73.45]
Is high fat diet a risk factor for breast cancer	154	37.02	[32.37–41.67]
Is smoking a risk factor for breast cancer	327	78.61	[74.65–82.56]
Is alcohol consumption a risk factor for breast cancer	261	62.74	[58.08–67.40]
Is first child at age more than 30 a risk factor for breast cancer	144	34.62	[30.03–39.20]
Is menarche below 11 years a risk factor for breast cancer	105	25.24	[21.05–29.43]
Is late menopause a risk factor for breast cancer	144	34.62	[30.03–39.20]
Is stress a risk factor for breast cancer	151	36.30	[31.66–40.93]
Is larger breast a risk factor for breast cancer	57	13.70	[10.39–17.02]
Does breast feeding decrease risk of breast cancer	317	76.20	[72.10–80.31]
Is painless breast lump a risk factor for breast cancer	267	64.18	[59.56–68.80]
Is null parity a risk factor for breast cancer	165	39.66	[34.95–44.38]
Is obesity a risk factor for breast cancer	140	33.65	[29.10–38.21]
Do oral contraceptive pills increase the risk of breast cancer	219	52.64	[47.83–57.46]
Is trauma to breast a risk factor of breast cancer	135	32.45	[27.94–36.96]
Does endogenous estrogen hormone increase the risk of breast cancer	234	56.25	[51.47–61.03]
Do you think giving births > 4 decreases risk of breast cancer	125	30.05	[25.63–34.47]
Does physical activity decrease the risk of breast cancer	158	37.98	[33.30–42.66]

### Attitude towards breast cancer

The attitude of the participants on breast lump was satisfactory, 342 **(**82.2%) of the participants were willing to be examined by a male doctor for their breast (data not shown). Similarly, 348 (83.7%) were willing to be examined by a doctor within one week suspecting of developing breast lump.

Regarding the first time diagnosis of breast cancer, the response of the participants was scared (61.8%), 95.7% consult a doctor, 10.1% use traditional medicine and 80.8% agree on Mastectomy (If necessary).

When the nurses were asked about their own overall risk of developing breast cancer, 22% thought that they were not at risk, 21.2% thought that they were at low risk, 11.1% at medium risk and 24% at high risk. The remaining 21.6% stated they don’t know.

Nurses were also asked if they had any of the risk factors for developing breast cancer, 51.0% answered they had none of the risk factors, 18.8% mentioned having one risk factor, 8.2% having two risk factors and 4.8% having three or more risk factors. On the potential risk factors of breast cancer,51.0% of the respondents answered none, while 18.8% mentioned are having one risk factor, 8.2% two risk factors and 4.8% three and more than three.

More than half (53.6%) of the participants believed that that breast cancer occurs more commonly in old women. In addition, 235 (56.5%) of the participants have thoughts that breast cancer is a curable disease. The overall understanding of the survival period (more than five years) after clinical diagnosed of having breast cancer was 59.4%.

### Knowledge about signs and symptoms of breast cancer

Table 3 shows that swelling or enlargement of the breast was the predominantly mentioned signs of breast cancer followed by pain or soreness of the breast. Similarly, more than 85% of the respondents stated that a lump in the breast, change in the size of the breast and discoloration/dimpling of the breasts are the major signs of breast cancer. Weight loss was the least (58.17%) mentioned sign and symptom of breast cancer.

**Table 3:** knowledge about the signs and symptoms of breast cancer among nurses in Asmara, Eritrea

Questions (Signs and symptoms mentioned)	Yes	%	95% Confidence Interval
Lump in the breast	358	86.06	[82.72–89.40]
Discharge from the breast	344	82.69	[79.05–86.34]
Pain or Soreness in the breast	368	88.46	[85.38–91.54]
Change in the size of the breast	358	86.06	[82.72–89.40]
Discoloration/dimpling of the breast	355	85.34	[81.93–88.75]
Ulceration of the breast	327	78.61	[74.65–82.56]
Weight loss	242	58.17	[53.42–62.93]
Changes in the shape of the breast	337	81.01	[77.23–84.79]
Inversion/ pulling in of nipple	288	69.23	[64.78–73.68]
Swelling or enlargement of the breast	374	89.90	[87.00–92.81]
Lump under armpit	319	76.68	[72.61–80.76]
Scaling / dry skin on nipple region	273	65.63	[61.05–70.20]

In addition, 56.5% of the participants thought that breast cancer is a curable disease. The overall understanding on the survival period (more than five years) after clinical diagnosis of having breast cancer was 59.4%.

### Knowledge and practice of nurses about screening methods

The knowledge of nurses on the correct age for initiation of breast self-examination was 75.8% and more than three quarters (80%) of them reported that they knew how to perform BSE and less than half of them (42%) knew the recommended frequency of conducting BSE.

Three-quarters (75.5%) of the nurses said they practiced BSE. Of these, 60.6% practiced once a month, 7.2% once every three months, 14.5% said they did BSE once a year, and 17.8% did not answer. The reasons given for not practicing BSE were; ‘carelessness’ (17.1%), ‘don’t have breast problem’ (14.2%), ‘unsure about its benefit’ (8.2), ‘don’t know how to do it’ (5.3), ‘too frequent to practice’ (2.9%), ‘don’t feel comfortable doing it’ (1.2%) and ‘don’t think I need to do it regularly’ (1%).

Thirty percent of the nurses practiced CBE. Of these, 14.9% were examined only once and 13.3% three times and above. The majority (55.3%) of those who did not practice CBE stated that they were reluctant because they had no signs and symptoms of breast cancer. Forty-seven nurses (11.3%) (37% of those aged > 40 years) had undertaken mammography examination in their life and only 8.7% of the overall study participants knew that 40 was the correct age for starting mammography as a screening method.

In this study, age and religious affiliation of respondents were associated with knowledge on signs and symptoms of breast cancer. Marital status was not associated with the KAP of nurses. Having breast problems was associated with the KAP of nurses whereas having a family history of breast cancer was only associated with the knowledge of risk factors. Nurses with bachelor’s degree had the highest knowledge compared to registered and associated nurses. The place of work was also significantly associated with the knowledge about breast cancer risk factors and its symptoms; nurses who worked at the Orotta Maternity Hospital had the highest knowledge (Table 4).

**Table 4:** Bivariate analysis of factors associated with KAP of nurses in Asmara, Eritrea

	N	Knowledge of Risk Factors (Mean ± SD)	Knowledge of Symptoms (Mean ± SD)	Knowledge and practice about breast cancer screening (Mean ± SD)
Age				
19–29	222	49.47 ± 19.06	83.68 ± 17.16	69.82 ± 17.06
30–39	65	50.26 ± 20.61	78.46 ± 18.47	69.91 ± 16.04
40–49	63	44.62 ± 23.44	78.52 ± 18.33	69.66 ± 21.23
> =50	66	48.52 ± 17.52	85.00 ± 20.62	68.52 ± 19.35
*p value*^*2*^		*0.333*	*0.042*	*0.961*
Marital Status				
Single	212	49.32 ± 19.95	84.06 ± 16.91	71.23 ± 14.25
Ever married	202	49.60 ± 19.49	80.45 ± 19.24	68.40 ± 19.77
*p value*^*1*^		*0.889*	*0.050*	*0.103*
Religion				
Muslim	35	48.71 ± 16.72	72.29 ± 17.17	66.35 ± 15.82
Christian	379	48.73 ± 20.09	83.23 ± 18.08	69.96 ± 13.55
*p value*^*1*^		*0.995*	*0.001*	*0.138*
Family Hx of Breast Cancer				
Yes	30	61.48 ± 15.64	76.87 ± 23.48	65.65 ± 18.29
No	384	47.71 ± 19.78	82.75 ± 17.69	70.02 ± 17.88
*p value*^*1*^		*0.001*	*0.080*	*0.180*
Having breast problem				
Yes	20	59.88 ± 8.85	73.00 ± 19.49	58.88 ± 13.54
No	396	48.20 ± 20.03	82.77 ± 18.07	70.14 ± 17.95
*P Value*^*1*^		*0.014*	*0.019*	*0.006*
Professional Level				
Associate Nurse	203	45.78 ± 20.09	82.36 ± 17.31	66.50 ± 18.23
Registered Nurse	170	49.22 ± 19.57	82.26 ± 20.02	72.22 ± 17.65
BSN	43	60.34 ± 14.73	82.09 ± 15.21	73.90 ± 14.72
*P Value*^*2*^		*0.001*	*0.996*	*0.002*
Unite of Work				
Orotta MCH	38	63.60 ± 12.50	91.58 ± 10.27	71.35 ± 15.85
Private hospital	44	54.80 ± 20.60	86.59 ± 15.99	73.48 ± 11.55
Medical wards	162	49.06 ± 21.91	80.98 ± 24.09	68.38 ± 13.60
Orotta Medical Surgical	50	36.67 ± 15.71	80.60 ± 16.83	68.57 ± 12.44
Community hospitals	82	*45.66* ± *15.42*	76.71 ± 20.31	71.54 ± 14.83
Ophthalmic hospital	40	45.97 ± 19.97	75.25 ± 17.54	66.39 ± 13.19
*P Value*^*2*^		*0.001*	*0.001*	*0.093*

*1 = T-test, 2 = ANOVA*

Moreover, there was a significant association between the professional level of nurses and BSE practice (*p* < 0.001) in which registered nurses had the highest practice (82.40%), followed by associated nurses (74.4%) and bachelor science in Nursing (BSN) (55.80%) (data not shown).

## Discussion

There are four important findings in this study. Firstly, nurses’ knowledge about the possible risk factors of breast cancer is a concern since many established risk factors of breast cancer were mentioned by less than 50% of them. Secondly, nurses had a good knowledge of the signs and symptoms of breast cancer in which each sign or symptom was mentioned by more than half of them. Thirdly, the practice of breast cancer screening was low and lastly, a family history of breast cancer, having breast problems, the professional level of nurses and the unit where they work predicts the KAP of nurses about breast cancer.

In some studies elsewhere, nurses’ knowledge about the risk factors of breast cancer has been found to be low [14, 16, 20] whilst other studies have reported nurses’ knowledge about risk factors of breast cancer to be high [13]. The fact that there are many risk factors of breast cancer [5] and new factors have been found to be associated with breast cancer might have affected the knowledge of the nurses. The unit they work may influence their knowledge. In the present study, nurses who worked at Orotta Maternity Hospital had the highest knowledge about the risk factors of breast cancer while nurses who were working at Orotta surgical ward had the lowest knowledge. Therefore, efforts need to be taken to increase the knowledge of nurses about the risk factors of breast cancer. Short on-site courses prioritizing those nurses who work outside maternity units/wards and nurses with associate level could help to update their knowledge.

In line with other studies [11, 15, 20] nurses in this study were found to have a good knowledge of the signs and symptoms of breast cancer. Nurses who work in the Orotta Maternity Hospital had high (91.6%) knowledge about the signs and symptoms of breast cancer.

Knowledge and attitude should be accompanied by practice. A gap in practice was identified in this study in which nurses were found to practice breast cancer screening infrequently. Nurses in this study reported practicing CBE and mammography less often. Only 30% of the nurses practiced clinical breast examination. This was similar to the findings from one study in Nigeria [11] and lower than a similar study in the same country [13]. Of all the nurses, only (11.3%) had undertaken mammography examination. Mammography is the gold standard for early detection of breast cancer but is not recommended for countries with limited resources due to its cost and technical complexity [25]. The practice of BSE among the nurses in this study was lower than studies conducted elsewhere [16, 19] and higher than some other similar studies [18, 20].

There were some limitations in this study. Firstly, the practice of breast cancer screening was reported, but nurses may not adequately remember whether they have done breast cancer screening or not. Secondly, since our subjects were those nurses working in hospitals only, this result may not reflect the knowledge of nurses who work in health centers and small clinics. Despite these limitations, we discovered important gaps in KAP of hospital nurses about breast cancer.

## Conclusion

This was the first study to assess the KAP of nurses’ about breast cancer. Nurses were found to have good knowledge of the signs and symptoms of breast cancer but little knowledge about the risk factors of the disease. The practice of breast cancer screening among the nurses was low. Therefore, training programs to update nurses’ knowledge about the risk factors of breast cancer and practice of breast cancer screening could potentially help in the practice of healthy habits among the nurses. This, indirectly, could also help to increase the knowledge and practice of the public about breast cancer.

## Abbreviations

BSE: Breast Self-Examination
BSN: Bachelor of Science in Nursing
CBE: Clinical Breast Examination
HRM: Human Resource Management
KAP: Knowledge Attitude and Practice
